# Systemic dysregulation of apolipoproteins in amyotrophic lateral sclerosis serum

**DOI:** 10.1002/2211-5463.70232

**Published:** 2026-03-19

**Authors:** Finula I. Isik, Russell Pickford, Hannah C. Timmins, Olivier Piguet, Glenda M. Halliday, Matthew C. Kiernan, Woojin Scott Kim

**Affiliations:** ^1^ Brain and Mind Centre The University of Sydney Australia; ^2^ School of Medical Sciences The University of Sydney Australia; ^3^ Bioanalytical Mass Spectrometry Facility University of New South Wales Sydney Australia; ^4^ Neuroscience Research Australia Sydney Australia; ^5^ Faculty of Medicine and Health University of New South Wales Sydney Australia; ^6^ School of Psychology The University of Sydney Australia; ^7^ South Eastern Sydney Local Health District Australia

**Keywords:** Amyotrophic lateral sclerosis, apoB, apoE, apolipoproteins, lipids, lipoproteins

## Abstract

Amyotrophic lateral sclerosis (ALS) is a fatal neurodegenerative disorder characterized by progressive motor neuron degeneration. Increasing evidence implicates systemic lipid perturbation in ALS pathogenesis. However, the extent and nature of apolipoprotein changes underlying lipid perturbations in ALS remain largely unknown. To address this, we performed a comprehensive analysis of major apolipoproteins involved in lipid metabolism and examined their association with lipoprotein membrane lipids in sporadic ALS (*n* = 32) and age‐matched healthy controls (*n* = 32), using ELISA and liquid chromatography–mass spectrometry. Compared with controls, serum levels of apoB, apoCI, apoCII, apoCIII and apoE were significantly elevated in ALS, whereas apoAI and apoAII were unchanged. Distributional analyses demonstrated a relative decrease in apoAI and an increase in apoB in ALS, resulting in an elevated apoB/apoAI ratio, a marker of atherogenic risk, alongside a reduced apoAI/apoE ratio. Correlation analyses revealed strengthened interrelationships among apolipoproteins in ALS, suggesting altered regulatory coordination. At the lipid level, phosphatidylcholine (PC) was increased, whereas sphingomyelin (SM) was reduced in ALS serum. Notably, the strong associations of apoB to both PC and SM observed in controls were absent in ALS. Biomarker analyses identified apoE as the strongest discriminator between ALS and control groups. Collectively, these findings demonstrate a coordinated disruption of apolipoproteins and lipoprotein‐associated lipids in ALS serum, with likely functional consequences for lipoprotein metabolism. This study provides new insights into lipid dysregulation in ALS pathobiology and supports the emerging view that ALS encompasses not only neurodegenerative processes but also systemic metabolic reprogramming.

## Graphical abstract

Amyotrophic lateral sclerosis (ALS) is a fatal disease that damages motor neurons. This study found that people with ALS show significant changes in blood fats and the proteins that carry them. Several apolipoproteins were higher, lipid balances were altered, and normal protein–lipid relationships were disrupted. ApoE best distinguished ALS from healthy individuals. Overall, the results show broad lipid metabolism disruption in ALS.

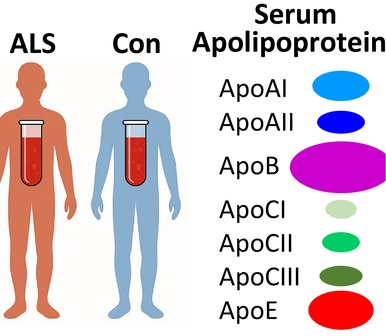

**Abbreviations**
ALS: Amyotrophic lateral sclerosis
apoAI: Apolipoprotein A‐I
apoAII: Apolipoprotein A‐II
apoB: Apolipoprotein B
apoCI: Apolipoprotein C‐I
apoCII: Apolipoprotein C‐II
apoCIII: Apolipoprotein C‐III
apoE: Apolipoprotein E
apoJ: Apolipoprotein J
AUC: Area under the curve
CNS: Central nervous system
CVD: Cardiovascular disease
ECDF: Empirical cumulative distribution function
FDR: Benjamini–Hochberg false discovery rate
HDL: High‐density lipoprotein
LDL: Low‐density lipoprotein
NfL: Neurofilament light chain
PA: Phosphatidic acid
PC: Phosphatidylcholine
PE: Phosphatidylethanolamine
PG: Phosphatidylglycerol
PI: Phosphatidylinositol
PS: Phosphatidylserine
ROC: Receiver operating characteristic
SM: Sphingomyelin
VLDL: Very low‐density lipoprotein

Amyotrophic lateral sclerosis (ALS) is a rapidly progressing neurodegenerative disease characterized by degeneration of motor neurons that results in muscle atrophy, gradual paralysis, and death usually within 2–5 years from diagnosis [1, 2]. Approximately 90% of ALS cases are sporadic, with remaining 10% associated with over 40 genes. ALS affects the entire human physiology, not just the central nervous system (CNS) [3], and increasing evidence indicates a strong link between lipid dysregulation and ALS, with changes in blood lipid levels and distribution contributing to disease processes [4]. Cross‐sectional and longitudinal studies of lipids in ALS serum show widespread changes in lipids affecting multiple pathways at different stages of the disease, implying dynamic metabolic remodeling rather than a single static signature [5].

The pivotal players of lipid metabolism in the blood are lipoproteins, which are ball‐like structures that carry and deliver various lipids from one part of the body to another. The general structure of lipoproteins is a layer of lipid membrane and a ‘cargo’ of lipids within, predominantly triglycerides and cholesteryl esters. The major lipids that make up the membrane are phosphatidylcholine and sphingomyelin, with lesser amounts of phosphatidylethanolamine and phosphatidylserine. Apolipoproteins are embedded in the membrane, and they control the trafficking and metabolism of lipoproteins. The most abundant lipoprotein in the human blood is low‐density lipoprotein (LDL). LDL carries a single molecule of apolipoprotein B (apoB) as its major protein component. The primary role of LDL is to transport cholesteryl esters from liver to peripheral tissues. The clinical significance of LDL is that elevated LDL levels are associated with cardiovascular disease (CVD). High‐density lipoprotein (HDL) is another prominent lipoprotein that contains apolipoprotein A‐I (apoAI) and lesser amounts of apolipoprotein A‐II (apoAII). ApoAI mediates the transport of HDL from arterial walls to liver, where cholesteryl esters are converted to bile acids and excreted from the body, reducing the risk of CVD [6]. The measurement of the apoB/apoAI ratio is commonly used to assess CVD risk, that is, the higher the ratio, the higher the CVD risk. In the context of ALS, apoAI was shown to be associated with lower risk of developing ALS [7].

Apolipoproteins C‐I (apoCI), apolipoprotein C‐II (apoCII), and apolipoprotein C‐III (apoCIII) are mainly present in very low‐density lipoprotein (VLDL). Of all the apolipoproteins, apolipoprotein E (apoE) is the most extensively studied in relation to both CVD and neurodegenerative diseases. ApoE plays a critical role in lipid metabolism throughout the body, including the CNS, and different apoE isoforms are associated with altered lipid transport and cognitive impairment. In fact, apoE is the strongest risk factor for late‐onset AD [8]. It is present predominantly in VLDL and to a lesser extent in certain subclasses of HDL. In the context of ALS, most studies show no robust, reproducible effect of apoE genotype on ALS risk [9].

Despite the critical importance of apolipoproteins in lipid metabolism in the periphery and the increasing recognition of their importance in the CNS, very little is known about how apolipoproteins are dysregulated in ALS, and how the interactions between apolipoproteins and lipoprotein membrane lipids are altered in ALS. Other than apoAI, apoB, and apoE, only scant information is known about the other apolipoproteins in the context of ALS. To address this knowledge gap, we investigated the full complement of apolipoproteins involved in lipoprotein metabolism and, in parallel, analyzed the major membrane lipid constituents of lipoproteins. We also investigated how apolipoproteins relate to neurofilament light chain. Finally, we analyzed the potential of apolipoproteins as biomarkers for ALS. Our study sheds new light on the understanding of lipid dysregulation underlying ALS pathobiology, reinforcing the concept that ALS involves not only neurodegenerative mechanisms but also systemic metabolic reprogramming.

## Materials and methods

### ALS patient and control serum

Individuals diagnosed with sporadic ALS [10, 11] (*N* = 32) were recruited from the Forefront ALS Clinic at the University of Sydney Brain and Mind Centre. Results were compared to healthy control volunteers [12] (*N* = 32) without neurological or psychiatric disorders or cognitive impairment. Blood samples were collected at the time of disease diagnosis. The study was approved by the University of New South Wales (approval number: HC12573) and the University of Sydney (approval numbers: 2012/160, 2014/539, 2017/928) human research ethics committees. All methods were carried out in accordance with the relevant guidelines and regulations, and that the study methodologies conformed to the standards set by the Declaration of Helsinki. Blood samples were obtained following written informed consent from the participant and/or primary carer as legal representative. All participants underwent a neurological examination, a comprehensive cognitive assessment and structural brain MRI, and met current consensus diagnostic criteria for ALS [13] or no neurological disease. Fasted blood samples (9 mL) were collected in tubes (BD Vacutainer SST II Advance Tube #367958), and serum prepared by centrifugation at 1000 ***g*** for 10 min at 4 °C, which was then aliquoted and stored at −80 °C until use.

### Apolipoprotein assays

ApoAI, apoAII, apoB, apoCI, apoCII, apoCIII, apoE, and apoJ in ALS and control serum were quantified using commercial human ELISA kits from Abcam (Melbourne, VIC, Australia) following the manufacturer's instructions (ApoAI catalog number #ab108803, dilution factor 1 : 100 000; apoAII, ab229423, 1 : 100 000; apoB, ab190806, 1 : 1000; apoCI, ab108808, 1 : 100; apoCII, ab168549, 1 : 200; apoCIII, ab154131, 1 : 4000; apoE, ab108813, 1 : 400; apoJ, ab1774447, 1 : 40 000). All apolipoproteins, except apoAII, were colorimetric kits and they read OD at 450 nm with a 570 nm correction. ApoAII utilized a fluorescence‐based assay (excitation/cutoff/emission: 530/570/590 nm). The apolipoprotein concentrations were interpolated from the standard curves using a four‐parameter logistic model (4PL, *R* ^2^ > 0.99).

### Neurofilament light chain assay

Frozen serum samples were thawed on ice and neurofilament light chain (NfL) was quantified using single‐plex R‐PLEX Human NfL Assay (#K1517XR‐2; Mesoscale Discovery, Rockville, MD, USA) following the manufacturer's instructions. The NfL signals were read using the Meso QuickPlex SQ120. A calibration curve (0–50 000 pg·mL^−1^) was generated using the NfL calibrator. Signal was fit to a 1/Y2‐weighted four‐parameter logistic (4PL, *R* ^2^ = 0.99991) to interpolate serum NfL concentrations.

### Chemicals and materials

Methanol, chloroform, and isopropanol were from Sigma Aldrich (St. Louis, MO, USA) and ultrapure water from Millipore (Bayswater, VIC, Australia). All solvents used were HPLC grade or higher. Glass pipettes and tubes were used wherever possible and the use of plasticware was minimized during lipid extraction to avoid contamination of samples. Glass tubes and glass transfer pipettes were purchased from Sigma (Bayswater, VIC, Australia) and VWR (Tingalpa, QLD, Australia). Lipid internal standards were purchased from Avanti Polar Lipids Inc (Alabaster, AL, USA). These include phosphatidylcholine (19 : 0), sphingomyelin (12 : 0), phosphatidylethanolamine (17 : 0), phosphatidylglycerol (17 : 0), phosphatidylserine (17 : 0), phosphatidic acid (17 : 0), phosphatidylinositol (17 : 0), cholesteryl ester (19 : 0) and triglyceride mix d5 (Avanti Code LM‐6000). Lipid internal standards were prepared as a mixture at 10 pmol·μL^−1^ in MTBE and methanol (1 : 1 v/v).

### Lipid extraction

Serum lipid extraction was based on the Bligh and Dyer method [14]. Briefly, serum samples were thawed on ice and 80 μL aliquots were transferred into glass tubes. Methanol (600 μL), chloroform (1000 μL), and ultrapure water (500 μL) were sequentially added with vortexing between each addition. Samples were then centrifuged at 1000 ***g*** for 10 min at room temperature. The lower solvent phase was collected and transferred to a new glass tube using a glass Pasteur pipette. Chloroform (600 μL) was added, vortexed, and centrifuged at 1000 ***g*** for 10 min. The lower phase was collected and combined with the first collected chloroform layer, and then the pooled extract dried under nitrogen gas. Dried lipid samples were reconstituted in 100 μL of isopropanol/methanol (1 : 1) and stored at −80 °C in glass LC–MS vials.

### Liquid chromatography—Mass spectrometry

Lipid extracts (10 μL) were analyzed using a Q‐Exactive HF Mass Spectrometer coupled to a U3000 UPLC system (ThermoFisher Scientific, Scoresby, VIC, Australia). Chromatography was performed at 60 °C on a Waters CSH C18 UHPLC column 2.1 × 100 mm, 1.8 μm with VanGuard guard column. Solvent A was 6 : 4 acetonitrile : water and Solvent B was 1 : 9 acetonitrile:isopropanol, both with 10 mm ammonium formate and 0.1% formic acid. Lipids were chromatographed according to the method of Castro‐Perez *et al*., [15]. Briefly, a 30‐min gradient running from 30 to 100% of solvent B was performed, eluting lipids in order of hydrophobicity. Column eluate was directed into the electrospray ionization source of the mass spectrometer where a HESI probe was employed. Source parameters were broadly optimized on a range of lipid standards prior to the analysis. The mass spectrometer was run in data dependent acquisition mode. A survey scan over the mass range 200–1200 at resolution 70 K was followed by 20 data dependent MS/MS scans on the most intense ions in the survey at 15 K resolution. Dynamic exclusion was used to improve the number of ions targeted. Cycle time was approximately 1 s. Samples were run in both positive and negative polarities. The samples were run in a random order (generated using microsoft excel). This is important to avoid batch effects/changing instrument performance effects. Data were analyzed in the lipidsearch software 4.2.29. Data were searched against the standard lipidsearch database with all common mammalian lipid classes included. The search results were then grouped according to sample type and aligned for differential analysis. Aligned data (containing lipid identity, peak area, retention time, etc.) were exported to excel. Abundance of lipids was obtained from peak areas for each lipid species and normalized between samples, to correct for batch effects from the sample preparation and the LC–MS/MS analysis, using the internal standards of the same lipid category.

### Statistical analysis

Serum concentrations of the eight apolipoproteins were compared between sporadic ALS patients (*n* = 32) and healthy controls (*n* = 32). Between‐group differences were assessed using Welch's two‐sample *t*‐test, and 95% confidence intervals for mean differences were computed using Satterthwaite's approximation. Nonparametric Mann–Whitney *U* tests were performed as a sensitivity analysis to account for potential deviations from normality. Effect sizes were estimated using Hedges' *g*, which corrects Cohen's *d* for small sample bias. To control for multiple comparisons across the seven apolipoproteins, *P*‐values from Welch's tests were adjusted using the Benjamini–Hochberg false discovery rate (FDR) procedure, with statistical significance defined as FDR‐adjusted *P* < 0.05. To visualize distributional differences beyond central tendency, empirical cumulative distribution function (ECDF) plots were generated for each apolipoprotein. Group medians were marked with vertical dashed lines. Individual data plots were generated using graphpad prism 10.6.1.

## Results

### Analysis of apolipoproteins in ALS serum

**Fig. 1:**
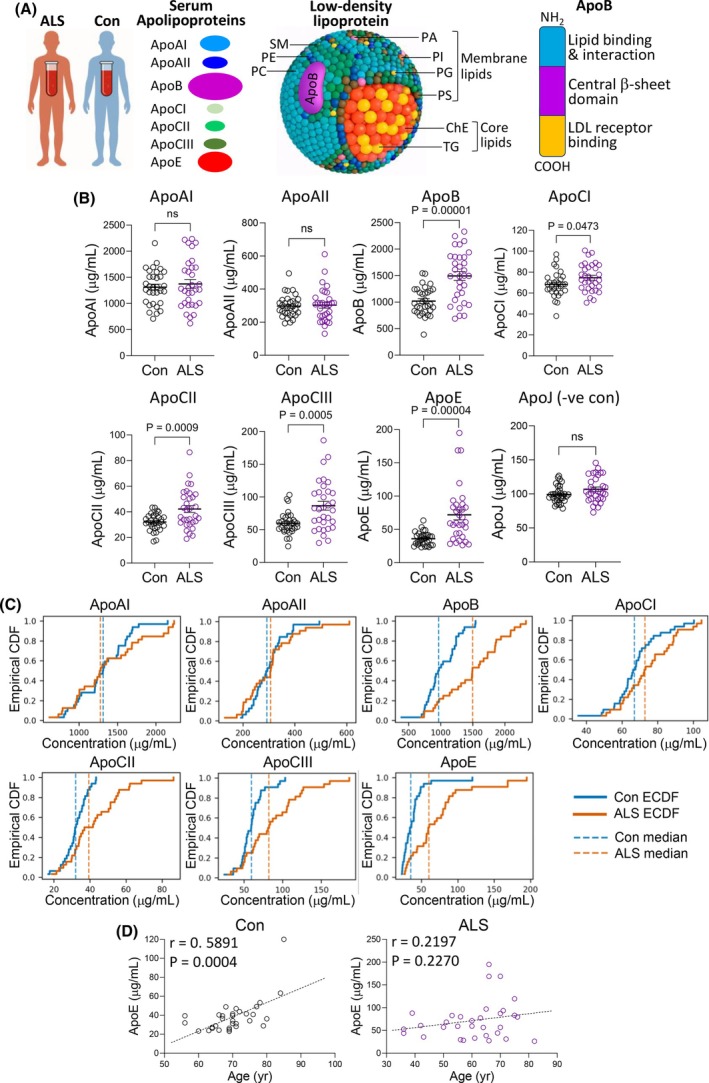
Analysis of apolipoproteins in amyotrophic lateral sclerosis and control serum. (A) Apolipoproteins in sporadic amyotrophic lateral sclerosis (ALS) (*N* = 32) and healthy control (Con) (*N* = 32) serum. Low‐density lipoprotein (LDL) with apoB and phosphatidylcholine (PC), sphingomyelin (SM), phosphatidylethanolamine (PE), phosphatidylserine (PS), phosphatidylglycerol (PG), phosphatidic acid (PA), phosphatidylinositol (PI), cholesteryl ester (ChE) and triglyceride (TG). Major domains of apoB. (B) Analysis of apolipoproteins in ALS and Con serum as measured by ELISA. Data represent mean and S.E.M. as error bars. Group differences were assessed using Welch's two‐sample *t*‐test. Not significant (ns) is defined as *P* > 0.05. (C) Apolipoprotein distributions of the two groups are visualized with empirical cumulative distribution function (Empirical CDF) plots. Group medians are marked with vertical dashed lines. (D) Pearson correlation analysis of apoE with age.

Apolipoproteins play a central role in lipid transport and metabolism in the circulation, and dyslipidemia is increasingly implicated in ALS pathogenesis. However, the full spectrum of apolipoproteins involved in lipid metabolism in ALS serum remains poorly characterized. We therefore undertook a comprehensive analysis of apoAI, apoAII, apoB, apoCI, apoCII, apoCIII, and apoE, as well as apoJ (nonlipoprotein apolipoprotein as a negative control) in sporadic ALS (*N* = 32) and healthy control (*N* = 32) serum (Fig. 1A). Five apolipoproteins showed significant differences after Benjamini–Hochberg false discovery rate (FDR) correction (FDR < 0.05) (Fig. 1B). ApoB was markedly elevated in ALS (mean = 1493.9 μg·mL^−1^) compared to controls (mean = 1020.5 μg·mL^−1^), with a mean difference of +473.4 μg·mL^−1^. ApoE was also significantly higher in ALS (71.9 vs 38.4 μg·mL^−1^; mean difference = +33.5 μg·mL^−1^). Similarly, apoCIII and apoCII were elevated in ALS. ApoCI showed a modest increase (*P* = 0.0473). No significant differences were observed for apoAI or apoAII. The negative‐control apoJ was unaltered in ALS, as expected.

Empirical cumulative distribution function (ECDF) plots (Fig. 1C) illustrated these differences across the entire distribution rather than isolated outliers. For apoB and apoE, ALS curves were consistently shifted to the right, indicating higher concentrations at all quantiles. ApoCII and apoCIII also demonstrated substantial rightward shifts, confirming elevated levels in ALS throughout the distribution. ApoCI exhibited only a modest separation, while apoAI and apoAII curves largely overlapped, reflecting similar distributions between groups. Vertical dashed lines marking group medians reinforced these patterns, with ALS medians notably higher for apoB, apoE, apoCII, and apoCIII. These ECDF patterns confirm that the observed differences represent systematic shifts in concentration profiles, supporting the robustness of the group differences identified by statistical testing. Finally, apoE was associated with age in controls, but not in ALS (Fig. 1D).

### Widespread apolipoprotein dysregulation in ALS serum

**Fig. 2:**
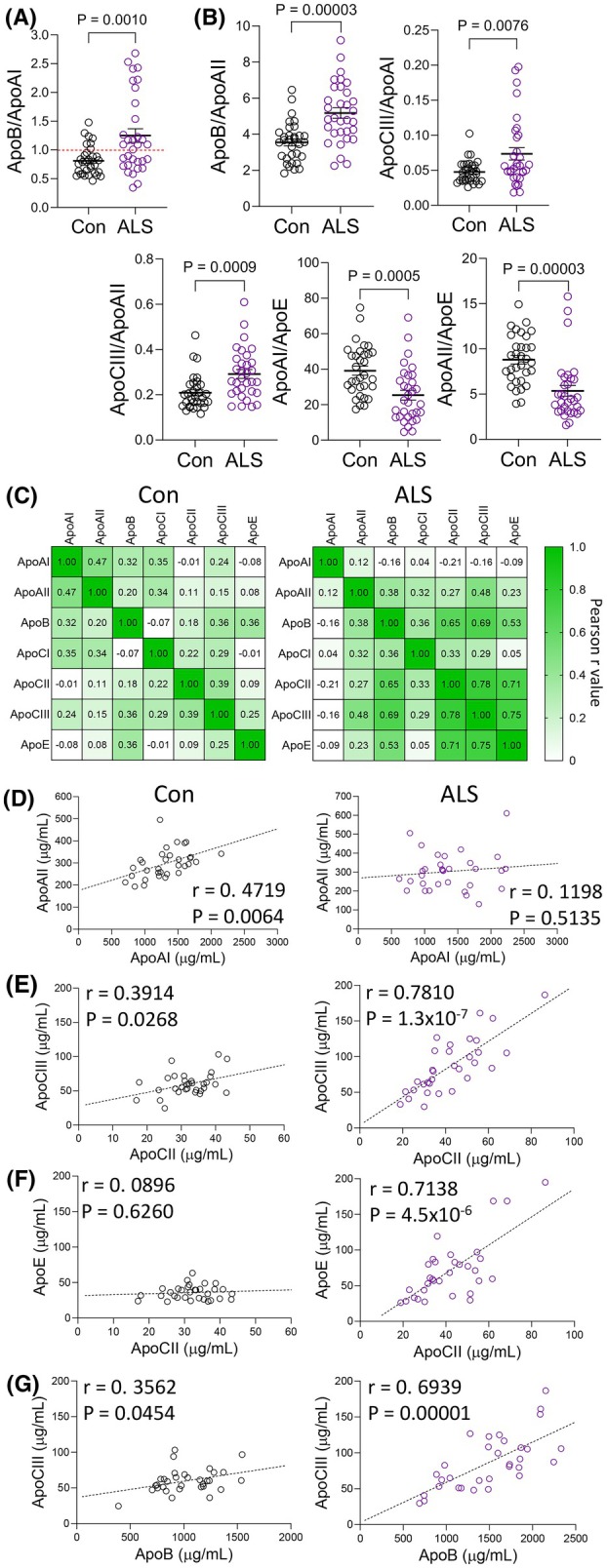
Analysis of inter‐relationship among apolipoproteins in amyotrophic lateral sclerosis serum. (A) Analysis of the apoB/apoAI ratio in sporadic ALS (*N* = 32) and healthy control (*N* = 32) serum. The red line denoting the threshold value of 1.0. Group differences were assessed using Welch's two‐sample *t*‐test. (B) Analysis of various ratios in ALS and control serum. Data represent mean and S.E.M. as error bars. (C) Pearson correlation coefficient (*r*) for all possible combinations of apolipoproteins. (D) Analysis of correlation between apoAI and apoAII. (E). Analysis of correlation between apoCII and apoCIII. (F) Analysis of correlation between apoE and apoCII. (G) Analysis of correlation between apoB and apoCIII.

To further understand the changes in apolipoproteins in ALS serum, we analyzed the ratio of apolipoproteins in ALS and control serum; altered apolipoprotein ratio is indicative of apolipoprotein dysregulation and/or metabolic disease state. The apoB/apoAI ratio, a common measurement of CVD risk [16, 17], was increased in ALS, with 53.1% of ALS patients being above the critical threshold value of 1.0 compared to only 18.8% for controls (Fig. 2A). The apoB/apoAII, apoCIII/apoAI, and apoCIII/apoAII ratios were also increased in ALS, whereas apoAI/apoE and apoAII/apoE were decreased in ALS (Fig. 2B). Second, we determined the Pearson correlation coefficient (r) for all possible combinations of apolipoproteins to quantify the strength of a linear relationship between two apolipoproteins in control and ALS serum (Fig. 2C). Notably, the association between apoAI and apoAII (both of which are present only on HDL) was strong in controls, whereas it was absent in ALS (Fig. 2D). The association between apoCII and apoCIII (both of which are present mainly on VLDL) was much stronger in ALS compared to controls (Fig. 2E). The association between apoE (present predominantly on VLDL) and apoCII was very strong in ALS, but absent in controls (Fig. 2F). Furthermore, the association between apoB (present on LDL and VLDL) and apoCIII, both of which are thought to be atherogenic, was stronger in ALS compared to controls (Fig. 2G). When put together, these results strongly suggest a widespread manifestation of apolipoprotein dysregulation in ALS serum.

### Disturbance of apolipoprotein–lipid relationship in ALS serum

**Fig. 3:**
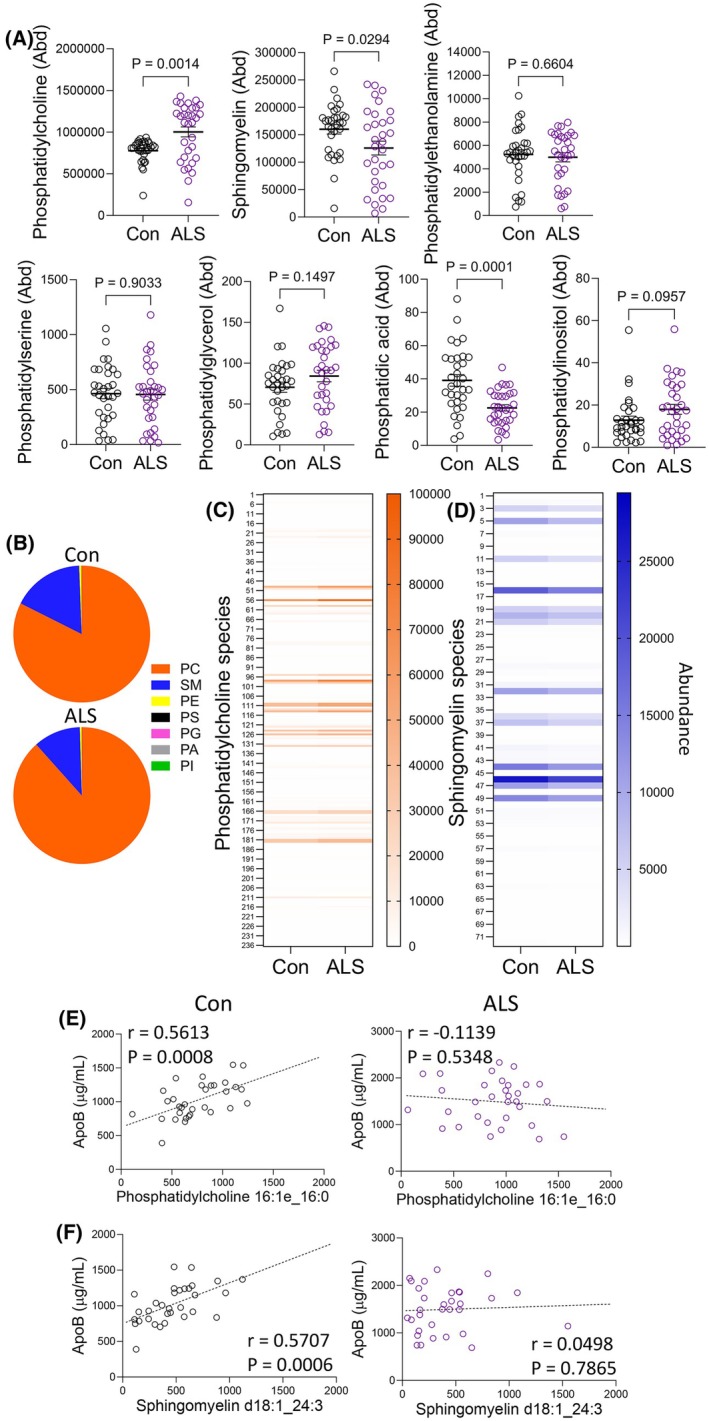
Analysis of lipoprotein membrane lipids in amyotrophic lateral sclerosis serum. (A) Analysis of the lipoprotein membrane lipids phosphatidylcholine (PC), sphingomyelin (SM), phosphatidylethanolamine (PE), phosphatidylserine (PS), phosphatidylglycerol (PG), phosphatidic acid (PA) and phosphatidylinositol (PI) in sporadic ALS (*N* = 32) and healthy control (*N* = 32) serum. Data represent mean and S.E.M. as error bars. Group differences were assessed using Welch's two‐sample *t*‐test. (B) Distributional analysis of the lipoprotein membrane lipids in serum. (C) A heatmap showing PC species in ALS compared controls. (D) A heatmap showing SM species in ALS compared controls. (E) Pearson correlation analysis of apoB with PC 16 : 1e_16 : 0. (F) Pearson correlation analysis of apoB with SM d18 : 1_24 : 3.

Apolipoproteins are embedded within the lipoprotein membrane, where their interactions with membrane lipids critically regulate lipoprotein structure and metabolic function; however, whether these interactions are preserved in ALS serum is unknown. We therefore conducted a comprehensive analysis of membrane lipid composition in ALS and control serum (Fig. 1A), using liquid chromatography‐mass spectrometry and LipidSearch software, in the same samples as those analyzed for apolipoproteins. The seven major membrane lipids—phosphatidylcholine (PC), sphingomyelin (SM), phosphatidylethanolamine (PE), phosphatidylserine (PS), phosphatidylglycerol (PG), phosphatidic acid (PA), and phosphatidylinositol (PI)—were identified (Fig. 3A). PC, the most abundant lipoprotein membrane lipid, was significantly higher in ALS than in controls. SM, the second most abundant lipoprotein membrane, was significantly lower in ALS. For the remaining lipids—PE, PG, PI, PS—no FDR‐significant differences were observed. Effect sizes for these were small (Hedges' *g* ≤ 0.42).

Consistent with these findings, the relative distribution of PC was increased in ALS serum, whereas SM distribution was decreased (Fig. 3B). In contrast, the distributions of other lipid classes were only modestly altered (Fig. 3B). Given the predominance of PC in lipoprotein membranes, this lipid class was examined in greater detail. A total of 236 distinct PC species were detected in serum, the majority of which were significantly elevated or exhibited a trend toward increased abundance in ALS (Fig. 3C). By comparison, 72 SM species were identified, some of which were significantly reduced in ALS (Fig. 3C). To assess the relationship between membrane lipids and apolipoproteins, Pearson correlation analyses were performed between individual PC and SM species and all apolipoproteins analyzed. In control serum, both PC and SM species were selectively associated with apoB, with PC 16 : 1e_16 : 0 (Fig. 3D) and SM d18 : 1_24 : 3 (Fig. 3E) exhibiting the strongest correlations. Notably, these lipid–apoB associations were absent in ALS serum (Fig. 3E,F), indicating a disruption of normal lipid–apolipoprotein coupling in ALS.

### Apolipoproteins as potential biomarkers for sporadic ALS

**Fig. 4:**
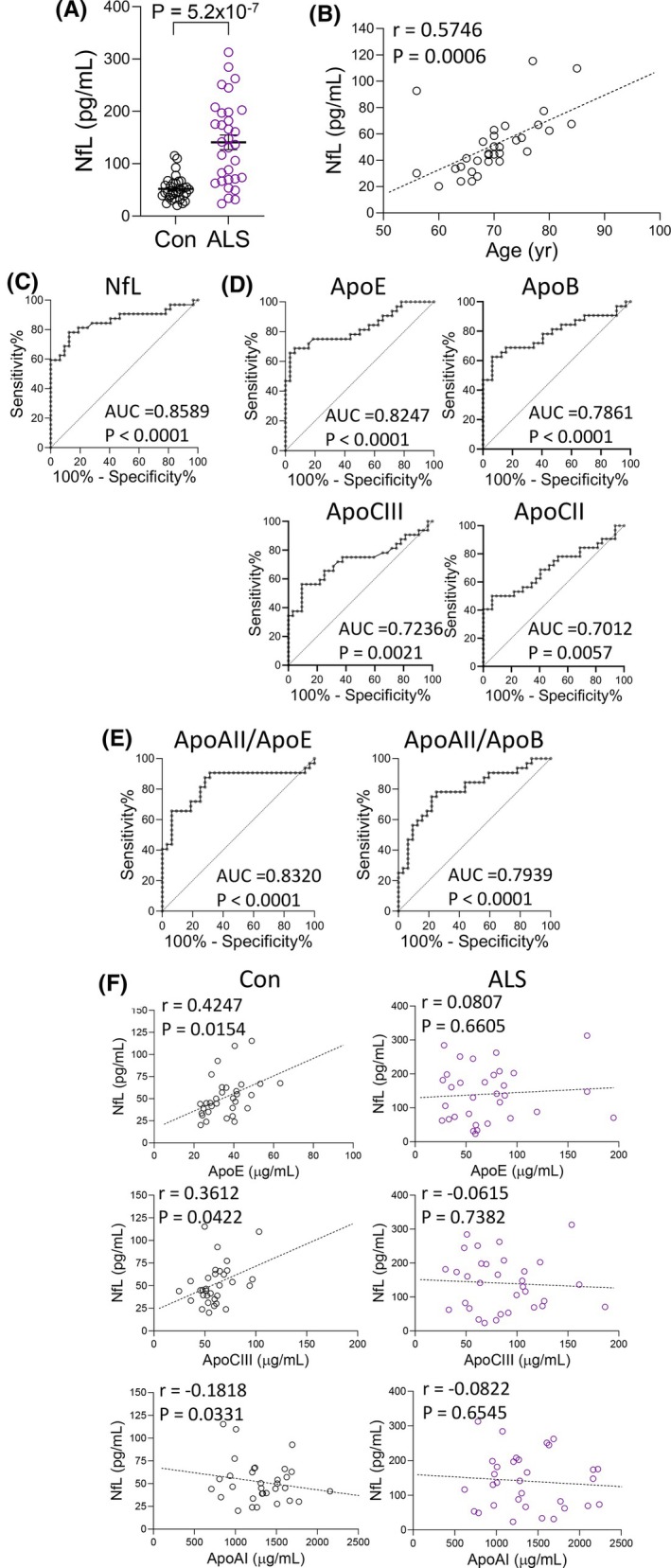
Evaluating apolipoproteins as potential biomarkers for sporadic ALS. (A) Measurement of neurofilament light chain (NfL) in sporadic ALS (*N* = 32) and healthy control (*N* = 32) serum. Data represent mean and S.E.M. as error bars. Group differences were assessed using Welch's two‐sample *t*‐test. (B) Pearson correlation of NfL with age in healthy controls. (C) Receiver operating characteristic (ROC) curve and area under the curve (AUC) value for NfL. (D) ROC curves and AUC values for apolipoproteins. (E) ROC curves and AUC values for the apoAII/apoE and apoAII/apoB ratios. (F) Pearson correlation analysis of NfL with apolipoproteins.

A lack of reliable blood‐based biomarkers is hampering or delaying the correct diagnosis of ALS. At present, no definitive circulating biomarkers specific to ALS have been established, with neurofilament light chain (NfL) serving primarily as a non‐specific marker of neurodegeneration. In light of this limitation, we explored the potential utility of apolipoproteins as candidate biomarkers for sporadic ALS. First, we confirmed that serum NfL levels were significantly elevated in ALS compared with controls (Fig. 4A), and NfL exhibited a positive association with age in healthy controls (Fig. 4B), consistent with previous reports [18, 19]. To evaluate the diagnostic performance of apolipoproteins, receiver operating characteristic (ROC) curve analyses were performed and area under the curve (AUC) values were calculated for each apolipoprotein, alongside NfL. The AUC for NfL was 0.8589 (Fig. 4C). Among the apolipoproteins, apoE emerged as the strongest discriminator between ALS and control groups (AUC = 0.8247), followed by apoB, apoCIII, and apoCII (Fig. 4D). In addition, analysis of apolipoprotein ratios revealed that apoAII/apoE and apoAII/apoB provided strong discriminatory performance (Fig. 4E). We further examined the relationship between apolipoproteins and neurodegeneration by assessing Pearson correlation between individual apolipoproteins and NfL in control and ALS serum. In controls, apoE and apoCIII showed positive associations with NfL, whereas apoAI was inversely associated with NfL (Fig. 4F). In contrast, no significant associations between apolipoproteins and NfL were observed in ALS serum (Fig. 4F).

## Discussion

The present study provides compelling evidence for pronounced and selective alterations in apolipoprotein abundance and distribution in the serum of individuals with sporadic ALS. In line with the growing recognition of systemic lipid dysregulation in ALS, we observed significant elevations in apoB, apoCI, apoCII, apoCIII, and apoE, whereas levels of apoAI and apoAII remained unchanged. Importantly, apoJ, included as a nonlipoprotein control, was also unaltered, underscoring the specificity of the observed apolipoprotein perturbations. ALS serum was further characterized by an increased apoB/apoAI ratio and a reduced apoAI/apoE ratio, indicative of a shift toward an altered and potentially dysfunctional lipoprotein profile. At the lipid level, ALS was associated with an increased PC content within the lipoprotein membrane, accompanied by a disruption of the normal association between apoB and PC. Together, these findings demonstrate coordinated but selective disturbances in lipoprotein‐associated apolipoproteins and membrane lipid composition in ALS. This integrated dysregulation strongly suggests impaired lipid transport and altered metabolic homeostasis in the ALS disease state, extending current models of ALS pathobiology beyond neuron‐centric mechanisms to include systemic lipoprotein dysfunction.

The observed elevations in apoB‐containing and apoE‐containing lipoproteins point to enhanced VLDL and LDL activity, potentially reflecting systemic metabolic stress.

Previous studies have shown that ALS patients often exhibit hypermetabolism and dyslipidemia [20, 21]. ApoB, the principal apolipoprotein of LDL, has been associated with altered energy utilization and represents an adaptive response to increased energy demands in ALS [22] as motor neurons and skeletal muscle have high energetic needs. Systemic lipid shifts—increased lipid mobilization or altered lipoprotein composition—could represent compensatory responses to maintain energy supply, or maladaptive lipid wasting that worsens disease. In an animal model of ALS, changes in lipid utilization contributed to diminished membrane repair function in the skeletal muscle. In Alzheimer's disease (AD) mouse models, high levels of apoB caused increases in neuronal degeneration [23]. One theory is that high levels of apoB could trigger inflammatory reactions in the arterial wall, facilitating atherosclerosis that impedes the energy supply to the brain [24, 25].

Meta‐analysis and Mendelian‐randomization studies have tested genetically instrumented lipid traits against ALS, and results converge on higher apoB being associated with increased ALS risk [26]. However, in the period after ALS diagnosis, apoB was shown to be associated with a lower risk of death [27]. These findings highlight the hypothesis that apoB biology plays an important role in ALS and that the relationship between apoB and ALS is complex and could be subject to potential confounding factors. In one experimental study, treating mice with CSF apoB from sporadic ALS patients induced motor dysfunction, TDP‐43 pathology, and neurodegeneration, suggesting a possible pathogenic role for apoB in ALS [28]. Furthermore, selective depletion/filtration of apoB from ALS CSF attenuated these effects [28]. These results provide mechanistic proof‐of‐principle that apoB (or apoB‐containing particles) could be neurotoxic in ALS and suggest CSF‐targeted removal as a possible therapeutic strategy.

Similarly, the marked elevation of apoE in ALS serum supports its role as a multifunctional lipid transporter. Dysregulated apoE metabolism has been implicated in several neurodegenerative diseases, including AD [29]. ApoE is a central modulator of AD risk and progression, with the ε4 isoform being the strongest common genetic risk factor for late‐onset AD and is associated with earlier onset, greater amyloid burden, and worse clinical progression [30]. However, most large clinical genetic/phenotype studies do not support a simple, reproducible effect of apoE on ALS risk. Also, apoE is not related to disease duration or overall survival [4]. While it influences cognitive phenotype or overlaps with frontotemporal dementia features in some ALS patients, it is not a clear ALS risk allele like it is for AD [31]. Overall, apoE was not related to disease duration or overall survival [4]. In ALS, apoE upregulation reflects a compensatory mechanism in response to increased lipid turnover. Interestingly, our findings revealed that apoE was associated with age in healthy controls, but not in ALS, suggesting that disease‐related mechanisms override typical age‐dependent lipid regulation.

The increases in apoCI, apoCII, and apoCIII further reinforce the theory of lipoprotein remodeling in ALS. ApoCII serves as an essential activator of lipoprotein lipase, facilitating triglyceride clearance, whereas apoCIII inhibits this enzyme, leading to impaired lipid metabolism. Their concurrent elevation in ALS serum could therefore indicate a dysregulated balance between lipid mobilization and storage, possibly contributing to the metabolic inefficiency observed in ALS patients. Collectively, these apolipoprotein alterations underscore a systemic disturbance of lipid and energy homeostasis in ALS. Such changes arise from increased lipid turnover secondary to neuronal degeneration or reflect a systemic metabolic adaptation.

Although the levels of apoAI and apoAII (both of which are on HDL) were not altered in ALS, their distributions in serum were significantly reduced in ALS. A prospective analysis of the UK Biobank (~ 502 409 participants with baseline blood tests, median follow‐up ~ 12 years) revealed that higher apoAI and HDL before disease onset were associated with lower risk of later ALS [7]. Consistent with this, an *in vitro* study showed that apoAI promoted endothelial survival in an ALS model, suggesting potential protective vascular and/or anti‐inflammatory roles for apoAI [32]. Therefore, apoAI could be a therapeutic target for ALS.

The apoB/apoAI ratio is widely used to assess CVD risk. ApoB is the main apolipoprotein of the atherogenic lipoproteins LDL and VLDL, whereas apoAI is the principal apolipoprotein of the anti‐atherogenic HDL. The apoB/apoAI ratio quantifies the balance between harmful and protective lipoproteins, and the higher the ratio the greater the CVD risk. Direct measurements of the apoB/apoAI ratio in ALS cohorts are scarce, with most clinical studies reporting apoAI and apoB separately. The expected pattern is that a higher apoB/apoAI ratio would correlate with greater ALS risk or worse prognosis, but that expectation is inferred from separate lines of evidence rather than demonstrated by large, dedicated ratio analyses. Consistent with this expectation, we observed that the apoB/apoAI ratio was elevated in ALS. Likewise, the Swedish AMORIS Cohort study reported that the ratio was associated with a higher incidence of ALS [33]. In the same study, the ratio was shown to be strongly associated with future myocardial infarction, stroke and major adverse cardiovascular events, with higher ratios predicting greater risk [34].

We also addressed the current lack of specific biomarkers for sporadic ALS and generated ROC curves for each of the seven apolipoproteins. We confirmed that serum NfL levels are significantly higher in ALS and increase with age in healthy controls. Among apolipoproteins, apoE showed the strongest discriminatory power (AUC = 0.8247), comparable to NfL (AUC = 0.8589). When evaluating apolipoprotein ratios, the apoAII/apoE ratio was particularly effective at differentiating ALS from controls, with AUC = 0.8320. The correlation analyses revealed that in controls, apoE and apoCIII were positively correlated with NfL, whereas apoAI showed an inverse relationship with NfL. In contrast, no apolipoprotein–NfL correlations were observed in ALS serum, suggesting, possibly, a disease‐specific disruption in apolipoprotein–neurodegeneration associations. There are no other reports of linkage between apolipoproteins and NfL in the context of ALS. In fact, very little is known about such linkage in neurodegenerative diseases, other than apoAI being inversely associated with NfL in multiple sclerosis [35]. Overall, our findings highlight disruptions in apolipoproteins potentially associated with neurodegeneration and provide a new scope for biomarker development for ALS.

Several limitations should be considered when interpreting our findings. This study included ALS patients and healthy controls of Caucasian ancestry, and lipid profiles differ across ethnic groups. For instance, a meta‐analysis reported lower triglyceride and HDL levels in Asian ALS cohorts compared with controls [36], suggesting that ethnicity influences lipid‐related outcomes. Additionally, confounding factors such as statin use, body mass index, comorbid cardiometabolic disease, and lifestyle variables could affect apolipoprotein and lipid levels, potentially biasing observed associations if not adequately controlled. Discrepancies among studies also reflect differences in study timing, as some investigations assess apolipoprotein and lipid changes before symptom onset (premorbid), while others focus on diagnostic or late disease stages. Given that apolipoprotein and lipid alterations precede clinical symptoms, cross‐sectional analyses could also be difficult to interpret, such that it is not easy to distinguish whether the changes observed are causal factors or consequences of ALS progression. To address these uncertainties, large‐scale, standardized, longitudinal studies in newly diagnosed and presymptomatic (at‐risk) cohorts are needed to clarify the temporal and mechanistic relationships between lipid metabolism and ALS pathogenesis.

Despite the limitations of our study, our findings support several focused mechanistic hypotheses for future studies. The elevated apoB and increased apoB/apoAI ratio suggest a shift from HDL‐mediated lipid recycling toward apoB‐containing lipoprotein export, potentially reflecting altered hepatic VLDL production or impaired HDL maturation in response to systemic energy stress. The marked increase in apoE raises the possibility of dysregulated apoE‐dependent lipid trafficking between periphery and CNS, potentially affecting membrane repair or blood–brain barrier lipid flux in motor neurons. The loss of normal apoB associations with PC and SM suggests structural remodeling of apoB particles, possibly due to altered phospholipid transfer, lecithin–cholesterol acyltransferase activity, or sphingolipid metabolism. Finally, reduced SM alongside increased PC supports a shift in membrane lipid remodeling—potentially via enhanced sphingomyelinase activity—that could disrupt membrane microdomains and promote pro‐apoptotic signaling. Together, these data point to coordinated disturbances in hepatic lipoprotein production, systemic lipid transport, and membrane lipid homeostasis as mechanistic contributors to ALS pathobiology.

In summary, the pathophysiological relationship between apolipoproteins and ALS remains incompletely understood. In this study, we systematically analyzed the major apolipoproteins, in conjunction with lipoprotein membrane lipids, in serum from ALS patients and healthy controls. Our findings revealed distinct biochemical alterations in lipoprotein composition in ALS and identified specific apolipoproteins with potential value as candidate biomarkers for disease detection. Furthermore, we explored the mechanistic implications of these alterations, proposing that dysregulated apolipoprotein metabolism contributes to the pathogenic processes underlying ALS. These results reveal new insights into the role of lipid dysregulation in ALS pathobiology and reinforce the concept that ALS involves not only neurodegenerative mechanisms but also systemic metabolic reprogramming, particularly within lipoprotein and lipid transport pathways. Future investigations should determine whether the observed serum apolipoprotein changes reflect central nervous system lipid disturbances or represent peripheral compensatory responses to neurodegeneration. Comprehensive, longitudinal studies integrating metabolomic, lipidomic and proteomic approaches will be essential to elucidate the mechanistic interplay between lipid metabolism and ALS progression, and to evaluate the potential of key apolipoproteins, such as apoE and apoB, as reliable biomarkers or therapeutic targets in ALS.

## Conflict of interest

The authors declare no conflict of interest.

## Author contributions

WSK contributed to the conception and design of the study. FII, RP, HCT, OP, GMH, MCK, and WSK contributed to the acquisition and analysis of data. WSK wrote the manuscript.

## Data Availability

All data supporting the findings of this study are included in this published article.
